# Exposure to the troubles in Northern Ireland, memory functioning, and social activity engagement: results from NICOLA

**DOI:** 10.1007/s10433-022-00683-5

**Published:** 2022-02-10

**Authors:** Joanna E McHugh Power, Joanne Feeney, Elizabeth Fowler, Alan J. McMichael, Philip Hyland, Brian A. Lawlor, Sharon Cruise, Claire Potter, Ian Young, Bernadette McGuinness, Frank Kee

**Affiliations:** 1 Department of Psychology, Maynooth University, Education House, Co Kildare, Republic of Ireland; 2 Centre for Public Health, Queen’s University Belfast, Belfast, Northern Ireland; 3 School of Medicine, Trinity College, Dublin, Republic of Ireland

**Keywords:** Traumatic exposure, Cognitive ageing, Cohort studies, Structural equation modelling

## Abstract

We investigated the potential impact of a cohort traumatic exposure, the Troubles in Northern Ireland, on memory functioning in later life, and the potential moderating effect of social activity engagement. Using data from 6571 participants aged 60 + in the Northern Ireland Cohort for the Longitudinal Study of Ageing (NICOLA) cohort, we used a structural equation modelling framework to explore associations between traumatic exposure during the Troubles and memory functioning. As expected, social activity engagement was positively associated with memory functioning, *β* = .102. Traumatic exposure was also positively associated with memory functioning, *β* = .053. This association was stronger at low levels of social activity engagement; among those with higher levels, there was little association, interaction *β* = − 0.054. The positive association between traumatic exposure during the Troubles and memory functioning was not moderated by the age at which the exposures occurred (based on analysis of a subsample with available data), interaction *β* = − 0.015. We conclude that superior memory functioning was associated with higher levels of traumatic exposure during the Troubles, particularly among those with lower levels of social activity engagement, and regardless of the age at which the exposures occurred. Future longitudinal analyses are required to build on these results, which potentially have implications for life-course epidemiology, in relation to critical periods for traumatising experiences.

**Supplementary Information:**

The online version contains supplementary material available at 10.1007/s10433-022-00683-5.

## Introduction

It is undisputed that age adversely impacts cognitive functioning, yet cognitive outcomes in later life vary widely across individuals (Agrigoroaei and Lachman 2011). Efforts have been made to account for this variance, yielding evidence for explanatory effects of many modifiable lifestyle factors, such as exercise, smoking cessation, management of hearing loss (Livingston et al. 2017) and social activity engagement (Fratiglioni et al. 2004; McHugh Power et al. 2016; Zunzunegui et al. 2003; Kelly et al. 2017). Modification of such factors may be particularly critical among older adults with non-reversible risk factors for cognitive decline, such as APOE-ε4 genotype (Henderson et al. 1995). Social activity engagement in particular has a strong evidence base connecting it to improvement in (or maintenance of) cognitive function in later life. As a corollary, social isolation is robustly associated with cognitive decline and dementia among older cohorts (Cacioppo and Hawkley 2009; Yu et al. 2020; Kuiper et al. 2015).

Another such non-modifiable risk factor may be exposure to psychological trauma during the lifespan. Such traumatic exposure in the general population is common, with cumulative prevalence estimated at 70% (Benjet et al. 2016). From a developmental systems perspective, traumatic exposures represent environmental contexts, and can account for individual variation in outcomes such as health and cognitive functioning (Molenaar et al. 2013). Empirical evidence exists for an association between traumatic exposure and cognitive dysfunction or decline, e.g. in first responders (Levy-Gigi et al. 2014), and among specific clinical populations such as older adults with anxiety disorders, and breast cancer survivors, and those with a history of childhood traumatic exposure (Petkus et al. 2018; Kamen et al. 2017). Exposure to the Holocaust was also found to be associated with worse cognitive functioning (Barel et al. 2010) HPA axis dysfunction (which could in turn cause cognitive dysfunction) (Yehuda et al. 2005), and increased incidence of dementia in later life (Kodesh et al. 2019), a finding interpreted with recourse to the vulnerability hypothesis–that exposure to genocide had left participants more susceptible to later-life morbidity.

These studies describe a negative impact of traumatic exposure upon cognitive outcomes in later life. However, others have argued that those who survive early life traumatic events represent a “survivor” cohort, and may possess trait resilience, which could be protective against future morbidities (Barel et al. 2010). Findings from the Irish Longitudinal Study on Ageing (TILDA) show that individuals who experienced childhood sexual abuse have superior cognitive functioning relative to those without such history (Feeney et al. 2013), and in the face of worse mental health. Similarly, Tedeschi and Calhoun’s post-traumatic growth concept (Tedeschi and Calhoun 2004) suggests that positive mental changes can occur as a consequence of trauma, and this concept may offset the deleterious impact of trauma on physical health (Greenblatt Kimron et al. 2019).

The ageing Northern Irish population have collectively been exposed to a higher-than-average number of traumatic experiences, due to the historical period of the Troubles, a period of 30 years of civil unrest which impacted almost everybody in the state (Muldoon et al. 2005). Traumatic exposure due to the Troubles are likely to have occurred during early adulthood for the majority of the current older population of Northern Ireland (NI), which may be a ‘critical period’, in the language of life-course epidemiology (Kuh et al. 2003), more proximally linked to cognitive decline in later life than childhood traumatic experience (Burri et al. 2013). The legacy of the Troubles may in part explain the increased rates of mental health difficulties found in NI relative to the Republic of Ireland (RoI) (O’Reilly and Stevenson 2003).

However, little empirical evaluation is available of the hypothesis that experience of the Troubles has an association with health outcomes. Additionally, little evidence is available in relation to the impact of traumatic events in later life (Cook and Simiola 2018), which is troublesome since older adults may be more likely to experience traumatic events (such as bereavement or serious illness) than younger adults (Ogle et al. 2014). However, it has also been suggested that time moderates the impact of trauma on aspects of cognitive function, such as autobiographical memory (Wittekind et al. 2017), which may mean that current older adults in NI are relatively unaffected by the traumatic experiences of the Troubles, which would have peaked more than 40 years ago.

To offset adverse associations between traumatic exposure and dementia risk, protective habits such as social and leisure activity engagement have been advised (Kodesh et al. 2019). Since traumatic exposure is a non-modifiable risk factor for dementia, encouraging those who have been exposed to trauma to engage in protective activities may help to lower their dementia risk. Social and leisure activity engagement has been linked to lower risk of dementia (Fratiglioni et al. 2004) and better cognitive function in later life (McHugh Power et al. 2016), and as such social activity interventions have been successfully implemented to improve cognitive function in later life (Pitkala et al. 2011). To corroborate this recommendation specifically for populations with traumatic exposure, it is necessary to explore potential moderating effects of social activity engagement on the association between traumatic exposure and outcomes of interest. Thus the current analysis tests the following hypotheses: that retrospective reporting of exposures to the Troubles is positively associated with later-life memory functioning, and, following recommendations made by Kodesh (Kodesh et al. 2019), that this hypothesised association is moderated by social activity engagement. Previous findings indicate that traumatic exposure is associated with domains such as processing speed and executive function (Petkus et al. 2018). We focus our analysis on memory functioning since this is a cognitive domain with a strong ageing component, and since it is also a key marker of early dementia.

## Methods

### Design & participants

Data were obtained from the NI Cohort for the Longitudinal study Of Ageing (NICOLA), a study of 8,450 community dwelling men and women aged 50 years and over, resident in NI. The Health and Social Care (HSC) Business Services Organisation (BSO) database in NI was used to prepare a sampling frame that comprised all private addresses containing one or more general practitioner (GP) registered adult aged 50 and over (people in care homes or other residential institutions were excluded at baseline). A systematic sampling process was then used to identify 14,492 addresses, ordered using geographic stratification, which were contacted with details of the study. Further information about these procedures can be found in the NICOLA Wave 1 key findings report (https://www.qub.ac.uk/sites/NICOLA/FileStore/Filetoupload,783215,en.pdf). Participants were included if they had the (self-assessed) capacity to provide informed consent. Overall sample size was calculated to be representative of the general Northern Irish ageing population. The NICOLA protocol incorporates three components. The first of these is a computer-assisted personal interview (CAPI), containing questions on the participants’ sociodemographic circumstances, their health (including physical and cognitive health, healthcare utilisation, health behaviours and use of medications), their employment circumstances, social connectedness and social participation. Wave 1 data collection took place between December 2013 and March 2016, and the CAPI was completed by 8,348 adults aged 50 and over. The second NICOLA component is a self-completion questionnaire (SCQ), which contains questions about the respondents’ participation in leisure activities, their mental and physical health, and their attitudes to risk. The SCQ also incorporated several questions about the participants’ experience of events related to the civil conflict in NI known as the Troubles (See Measures below), as well as other traumatic events and posttraumatic stress symptoms. Since not all participants who completed the CAPI went on to complete the SCQ, different sample sizes across the measures can be observed.

All individuals participating in the CAPI were also invited to undergo the third component of NICOLA data collection, a health assessment. This incorporated the collection of biological samples and genetic biomarkers; measures of cardiovascular function, respiratory function, physical activity, ophthalmology, and anthropometry; and several cognitive measures. The majority of the health assessments were conducted at the NI Clinical Research Facility at Belfast City Hospital, with a smaller number being conducted in the participants’ homes if it was impractical for them to travel. Informed written consent was collected from all study participants. The current analysis, focusing on later-life memory functioning, used data gathered only from those aged 60 and over (6571 participants).

### Measures

#### Social activity engagement

In order to create a measure of social engagement, a range of social activities were recorded from 5,965 participants. Overall, 38% (*n* = 2292) said that they participated in at least one social activity. Of these 2292, 30.5% (929) participated in hobbies and social clubs; 31% (712) participated in sports/exercise groups; 13.5% (309) participated in local community or neighbourhood groups; 5% (110) participated in groups for children or young people; 7.6% (175) participated in adult education groups; 12.8% (293) participated in groups for older people; 1.6% (36) participated in environmental groups; 6.8% (157) participated in health, disability, or welfare groups; 0.9% (22) participated in political groups; 0.8% (19) participated in trade union groups; and 34% (783) participated in religious groups. A count of involvement in all groups was created so that participants could have participated in a minimum of 0 and a maximum of 11 social activities (see Table 2, below).

#### Troubles exposure

Questions assessing participants’ experiences of the Troubles were adapted from the NI Poverty and Social Exclusion (PSE) study (more information at http://www.poverty.ac.uk/pse-research/legacies-troubles), 2012 version (see Table 1). Since associated subjective trauma was not reported, these items could instead be described as querying potentially traumatic events. Data were available for 3275 individuals. Questions on experiences during the Troubles were grouped in the SCQ into those about “death” (question 69; whether an individual experienced a close friend, relative, or other individual dying as a result of the Troubles), “injury” (question 70; whether an individual experienced a personal injury or the injury of a close friend, relative, or other individual as a result of the Troubles), “witness” (question 71; whether the individual witnessed a bomb explosion, murder, gunfire, rioting, or another individual being assaulted during the Troubles). These three groupings were specified as latent variables in an initial measurement model. A fourth grouping, “authorities” (questions 72–77; whether the individual had interactions with the authorities such that they were either sent to prison for Troubles-related activity, or had their home searched by police or army) was also specified in the model, but it was later removed because of poor loading. The measurement model (and all subsequent analyses) were conducted using the lavaan package in R Studio (Rosseel 2012). All questions in Table 1 were coded such that 0 indicated that this experience had not been reported by the respondent, and 1 indicated that it had been reported by the respondent. The model converged normally after 61 iterations. Fit was acceptable, *χ*^2^_51_ = 1412, CFI = 0.90, TLI = 0.87, RMSEA = 0.084 (CI_90_ = 0.080, 0.088), SRMR = 0.046. Factor loadings were all acceptable and above 0.4. There were significant covariances among the latent factors (0.881 between Death and Injury; 0.593 between Death and Witness; 0.689 between Injury and Witness, all significant at *p* < 0.001). A second measurement model with a higher-order latent factor (Troubles Exposure) was also tested but this did not converge. A third measurement model was conducted which included Memory as an additional latent factor, and this model is described in Table 2, below.

**Table 1:** Questions about participants’ experience of the Troubles

Q69. Thinking of the Troubles, did you experience any of the following? (Death)
(a) A close friend was killed
(b) A close relative was killed
(c) Someone else that you knew personally was killed
(d) No close friend or relative was killed
Q70. Again, thinking of the Troubles, did you experience any of the following? (Injury)
(a) I was physically injured
(b) A close friend was physically injured
(c) A close relative was physically injured
(d) Someone else you know personally was injured
(e) None of the above
Q71. Have you yourself directly witnessed any of the following events? (Witness)
(a) A bomb explosion
(b) A murder
(c) Gunfire
(d) Rioting
(g) Someone being assaulted
(h) Other serious violence
(i) None of the above
Q72. Have you or anyone you know spent time in prison because of the Troubles? (Authorities)
(a) Yourself
(b) Close friends
(c) Close relatives
(d) Other relatives
(e) Others
Q74. Did you ever have your house searched by the police or army? (Authorities)
Q75. How many times was your house searched? (Authorities)
Q76. Did you ever have to move house due to attack, intimidation, threats, or harassment? (Authorities)
Q77. Did you ever have to leave a job because of an attack, intimidation, threats, or harassment? (Authorities)

**Table 2:** Measurement Model (using maximum likelihood estimator and full information maximum likelihood for missingness) describing three first-order latent variables (Death, Injury, Witness)in relation to Troubles Exposure, and one latent Memory variable

	Factor Loading	Standard Error	Z	p	Percentage of individuals reporting (n in brackets)
Death					
Close Friend	0.719	0.007	40.581	< .001	17.26% (626)
Close Relative	0.424	0.006	22.361	< .001	10.47% (381)
Someone known personally	0.644	0.009	37.340	< .001	51% (1881)
Injury					
Self	0.410	0.004	23.028	< .001	4.47% (166)
Close Friend	0.757	0.006	46.175	< .001	19% (701)
Close Relative	0.524	0.006	29.839	< .001	14.38% (529)
Someone known personally	0.659	0.008	39.806	< .001	38.58% (1429)
Witness					
Bomb explosion	0.608	0.008	37.759	< .001	45.96% (1716)
Gunfire	0.727	0.007	47.398	< .001	36.99% (1377)
Rioting	0.733	0.008	48.063	< .001	44.6% (1662)
Someone being assaulted	0.734	0.007	47.538	< .001	24.06% (885)
Other Serious Violence	0.728	0.006	46.833	< .001	19.76% (721)
Memory					
Immediate Recall 1	0.818	0.020	72.364	< .001	
Immediate Recall 2	0.880	0.021	80.412	< .001	
Delayed Recall	0.852	0.027	76.837	< .001	
Animal Naming	0.510	0.121	23.516	< .001	

#### Memory

Immediate and Delayed Word Recall are tasks evaluating episodic memory adapted from the version used in the Health and Retirement Study. For the immediate recall task, one (of four) randomly selected list of ten words was read to the participants and participants were tasked with repeating these ten words, yielding a score out of 10. Immediately after this, participants were given a second opportunity to recall the same ten words, yielding a second score of Immediate Word Recall. After a delay, during which the participants answered the CAPI section on health, they then recalled the words for a third time, and their score out of 10 was taken as the Delayed Word Recall measure.

An “Animal Naming” task of verbal fluency was used to evaluate semantic memory. This task involves instructing participants to name as many animals as they can in a sixty-second timeframe. This task is also used in the Health and Retirement Study and other harmonised cohort studies of ageing. A latent “Memory” factor with four indicators (Immediate word Recall 1 and 2; Delayed Word Recall; Animal Naming) was tested using a second measurement model that also included the latent Troubles measures described above. A similar approach was previously taken by the authors using data from the Irish Longitudinal Study on Ageing (McHugh Power et al. 2019).

This model converged normally after 72 iterations and model fit was good, *χ*^2^_98_ = 1589, CFI = 0.939, TLI = 0.925, RMSEA = 0.049 (CI_90_ = 0.047, 0.052), SRMR = 0.046. All memory items loaded well (minimum factor loading of 0.510; see Table 2). There were significant correlations between Memory and Injury, *r* = 0.059, *p* = 0.011; and between Memory and Witness, *r* = 0.153, *p* < 0.001, but not between Memory and Death, *r* = 0.021, *p* = 0.400.

#### Covariates

Choice of covariates was based on the following principles (VanDerWeele 2019): control for covariates that are causes of the exposure, or of the outcome, or both (including proxies for unmeasured variables that cause both), except for those that qualify as instrumental variables. We could not find theoretical justification to expect predictors of traumatic exposure to the Troubles. We included, as known causes of memory decline, age in years, gender (male or female), education level (operationalized as an ordinal variable with three levels as per TILDA: no qualifications, second level qualification, third level or higher qualification), and depressive symptoms (a single item about frequency of feelings of depression, responded as a five point scale from none to extreme). For a subset of participants (*n* = 1713), information was available on a question about time of the worst events of the Troubles (“Thinking of the worst thing that happened to you because of the Troubles, when was this?”) with categorical responses (1969–1973; 1974–1978; 1979–1983; 1984–1988; 1989–1993; 1994–1998, after 1998). The middle year of each category was used to calculate an approximate age of each of 1713 participants during the worst thing that had happened to them because of the Troubles (see Table 3), and this was also included in the model as a covariate.

**Table 3:** Descriptive Statistics for the NICOLA sample (SD = standard deviation; frequency given instead of mean for categorical variables; n = sample available for given variable (dependent on whether participants completed the self-completed questionnaire as well as the health assessment)

	Mean	SD	Frequency (n in brackets)	Range	n
Current age	72.4	8.69		60–105	6571
Age during the (self-rated) peak of the troubles			19: 2% (34), 21: 3% (50), 26: 4.4% (75), 31: 7.7% (131), 36: 12.4% (213), 41: 31.2% (546), 46: 38.8% (664)	19–46	1713
Gender			Female: 54.1% (3554) Male: 45.9% (3017)		6571
Marital status			Married: 61.5% (4040) Living with partner: 2% (139) Single: 7.5% (491) Separated: 3.4% (222) Divorced: 7.1% (466) Widowed: 18.5% (1213)		6571
Education level			No qualification: 29.2% (1901) Second level qualification: 56.3% (3665) Third level qualification: 14.5% (944)		6510
Depressive symptoms			None: 60.4% (2338) Mild: 25.1% (970) Moderate: 11.3% (436) Severe: 2.6% (102) Extreme: .6% (25)		3871
Mini mental state examination	28.29	1.9		16–30	2767
Any social activity engagement –yes or no			No: 61.5% (4038) Yes: 38.5% (2533)		6571
Social activity engagement (Count of activities)	0.6	.98		0–9	6571
Immediate recall 1	5.73	1.74		0–10	5813
Immediate recall 2	7.49	1.9		0–10	5810
Delayed recall	6.15	2.4		0–10	5815
Animal naming	18.43	5.44		3–41	2655

### Data analysis

A structural equation modelling (SEM) framework, using the lavaan package in R software (Rosseel 2012) was used to explore the hypotheses that social activity engagement would moderate the association between Troubles Exposure on Memory. SEM is superior to the linear regression approach to moderation, in its management of nonlinear relationships within interactions and of measurement error, which is often exacerbated in the product terms used to evaluate interactions in regression models (McCallum et al. 2002). Evaluation of moderation effects between latent and observed variables is complex, and can be done using the calculation of product indicators, but this is not possible with binary indicators (Huang and Bentler 2009). As such, data pertaining to each of the three latent Troubles variables were instead used to create a count based on Q69, Q70 and Q71 respectively (see Table 1), yielding count scores of Death, Injury, and Witness. These three counts were then used as indicators for a latent “Troubles Exposure” variable in a measurement model (see Supplementary Materials 1), and in all subsequent analyses. First, a SEM was run with main effects only (“SEM without Moderation”). Product indicators were then calculated between Troubles Exposure and Social Activity Engagement (both were mean-centred beforehand), as per guidance given previously (Kenny and Judd 1984), which were then used as indicators for a latent interaction term between Troubles Exposure and Social Activity Engagement (“SEM with Moderation”). Bootstrapped standard errors (*n* = 1000) and maximum likelihood estimation was used throughout with a full information maximum likelihood approach to missingness, to allow the use of the maximum amount of data available. Confidence intervals were calculated for all parameters at the 95% level.

## Results

### Descriptive statistics

The sample are described in Table 3. All available data for all relevant variables was included at both descriptive and inferential stage of data analysis. As per the scores of the mini-mental state examination (Folstein et al. 1975) participants were broadly free of cognitive impairment.

#### SEM without moderation

An initial model was performed with main effects only and excluding the moderation effect to provide a baseline comparator. Bonferroni corrections for multiple comparisons, therefore, set alpha at 0.025. The model converged normally after 79 iterations and demonstrated acceptable fit, *χ*^2^_69_ = 1084, CFI = 0.94, TLI = 0.92, RMSEA = 0.061 (CI_90_ = 0.058, 0.064), SRMR = 0.077. Sample-size-adjusted Bayesian Information Criterion (BIC) was 202480, and Akaike Information Criterion (AIC) was 202310. Being younger, *β* = − 0.419, *p* < 0.001, male, *β* = 0.114, *p* < 0.001, having lower depression, *β* = − 0.119, *p* < 0.001, higher education, *β* = 0.254, *p* < 0.001, higher levels of Troubles Exposure, *β* = 0.048, *p* = 0.01, and higher levels of Social Activity Engagement, *β* = 0.102, *p* < 0.001, were all associated with higher levels of the latent Memory variable.

#### SEM with moderation

**Fig. 1:**
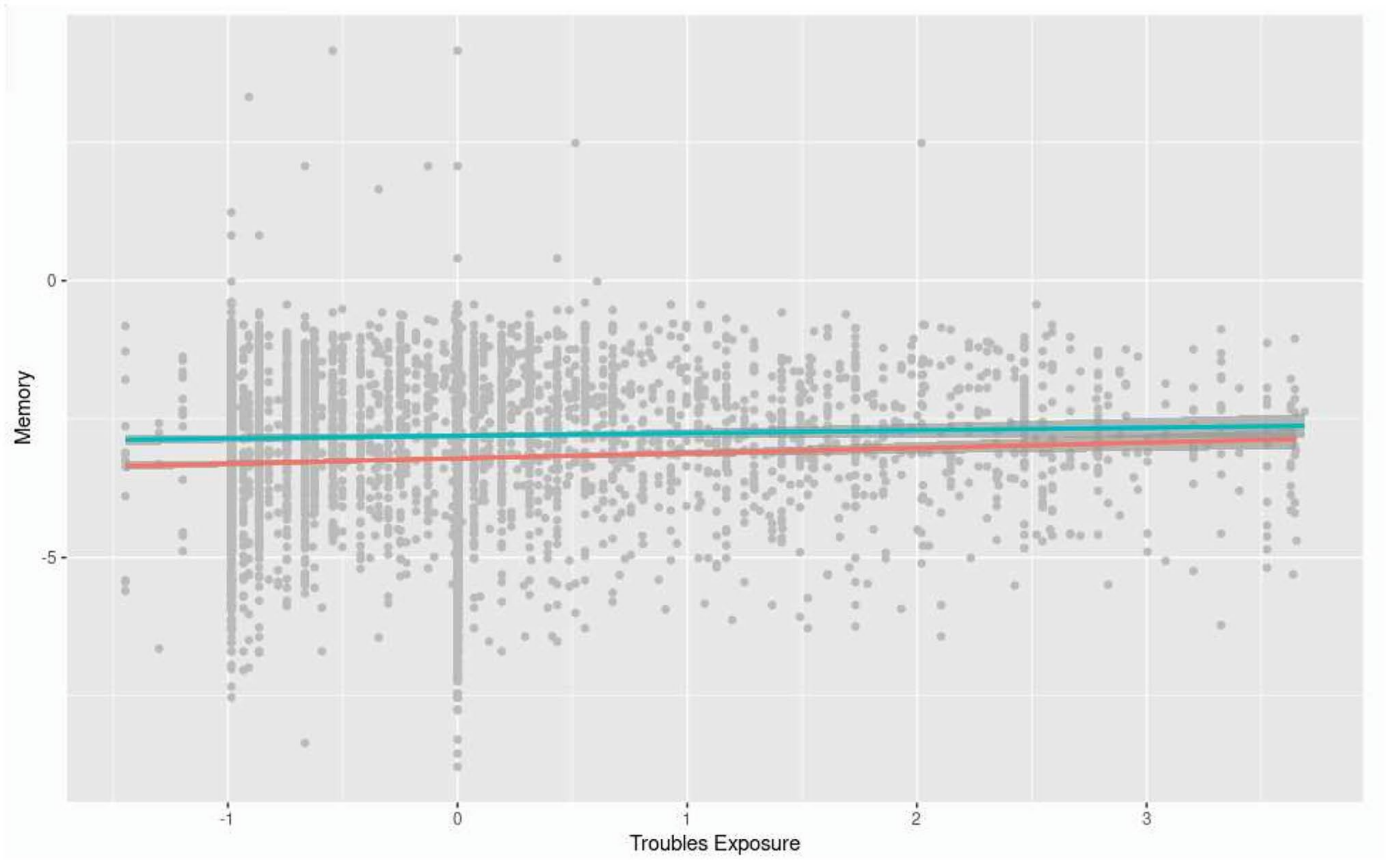
Moderation effect of Social Activity Engagement on effect of Troubles Exposure on Episodic Memory (red line represents low levels of social activity engagement, while green line represents high levels of social activity engagement)

The model converged normally after 78 iterations and demonstrated acceptable fit, *χ*^2^_77_ = 1272, CFI = 0.94, TLI = 0.92, RMSEA = 0.049 (CI_90_ = 0.046, 0.051), SRMR = 0.066. AIC was 232493 and sample-size-adjusted BIC was 232702. In the measurement component of the model, memory was included as a latent variable with four indicators, Immediate Recall 1 and 2, Delayed Recall, and Animal Recall, which had factor loadings of 0.826, 0.876, 0.858, and 0.530 respectively. Troubles Exposure was included as a latent variable with three indicators, Death, Witness, and Injury, all mean-centred prior to inclusion, and with factor loadings of 0.702, 0.873, and 0.638 respectively. An interaction product term was built as a latent factor, Interaction, with three indicators, Death, Injury, and Witness, each separately multiplied by Social Activity Engagement (mean-centred), which achieved factor loadings of 0.756, 0.658, and 0.816 respectively. Comparing AIC and sample size adjusted BIC between the model with moderation and the model without, fit was better in the version without (*χ*^2^ difference = 187.3, *p* < 0.001, df difference = 34). Memory was associated with being younger, *β* = − 0.42, *p* < 0.001, being male, *β* = 0.113, *p* < 0.001, greater education level, *β* = 0.253, *p* < 0.001, and lower depression, *β* = − 0.131, *p* < 0.001, higher Troubles Exposure, *β* = 0.053, *p* = 0.007, higher levels of Social Activity Engagement, *β* = 0.106, *p* < 0.001. The interaction term between Troubles Exposure and Social Activity Engagement was significant at *β* = − 0.054, *p* = 0.01 (see Table 4). A negative interaction term can be interpreted to mean that at higher levels of Social Activity Engagement, there is less of an effect of Troubles Exposure on Memory (see Fig. 1).

**Table 4:** Structural component of the SEM conducted with Memory as a (latent) criterion variable, Troubles Exposure as a (latent) exposure, and Social Activity Engagement as the moderator (betas are standardised estimates; standard errors are bootstrapped with 1000 draws)

Predictor	Beta	SE	Z	*P*	CI_95_
Age	− 0.420	0.002	− 27.692	< .001	− .064, − .056
Sex	0.113	0.030	9.255	< .001	.225 − .344
Depression	− 0.119	0.024	− 7.283	< .001	− .225–.138
Education	0.253	0.025	19.185	< .001	.436 − .539
Troubles Exposure	0.053	0.024	2.700	.007	.019 − .115
Social activity engagement	0.106	0.015	8.731	< .001	.101 − .161
Troubles Exposure * Social activity engagement	− 0.054	0.026	− 2.583	.010	− .120–.014

## Discussion

The current study investigated whether exposure to the Troubles would be associated with memory functioning in older NI adults, and whether this association would be moderated by social activity engagement. Better performance in memory tasks was associated with higher levels of Troubles Exposure, and with higher levels of social activity engagement. The association between Troubles Exposure and memory was also moderated by social activity engagement, such that at low levels of social activity engagement, those with higher levels of Troubles Exposure did better on memory, while at higher levels of social activity engagement there was little association between Troubles Exposure and memory.

The pattern of main effects found is compatible with previous findings. Social activities may constitute a cognitively stimulating pastime (Scarmeas and Stern 2003), which could bolster cognitive function in the ageing brain, according to the “use it or lose it” hypothesis of cognitive functioning (Hultsch et al. 1999). Our (positive) main effect of Troubles exposure on memory functioning is also compatible with findings reported by Feeney and colleagues based on data from TILDA (Feeney et al. 2013) which showed that retrospective reporting of childhood sexual abuse was associated with superior cognitive functioning performance in later life. This is also compatible with Tedeschi and Calhoun’s theory of post-traumatic growth, that positive changes can occur after trauma as well as negative ones (Tedeschi and Calhoun 2004). Feeney and colleagues had controlled for social activity engagement and found no effect–in our findings we report a main effect (positive) of social activity engagement and Memory, but also that the effect of Troubles Exposure is different at high and low levels of social activity engagement.

The finding that participants with high levels of Troubles exposure had good outcomes in relation to memory functioning is at odds with other findings in the literature, however, which points to traumatic exposure predicting cognitive decline (Kamen et al. 2017; Barel et al. 2010; Petkus et al. 2017). There are two possible, interesting explanations to this discrepancy. It should be noted that these previous studies explored associations between childhood trauma and later cognitive functioning, while in the current study (where participants currently have a median age of 71, and experienced the height of the Troubles around 40 years ago) we describe an effect of adulthood trauma on later memory functioning. It is possible, as previously suggested (Burri et al. 2013), that adulthood and childhood trauma exert effects of differing magnitude on later life memory functioning. Alternatively, the nature of the traumatising phenomenon may be different–the childhood trauma and genocide trauma described in these previous studies is inarguably of an entirely different nature and higher degree than the Troubles. It is possible that the degree of traumatisation yielded by the Troubles was sufficient to confer an inoculation effect without yielding a deleterious impact on the brain health of survivors. Childhood trauma may directly impact brain health by exerting a deleterious impact on the developing brain, as outlined in the traumagenic neurodevelopmental model of schizophrenia (Read et al. 2001). This explanation fails to account for the pattern of results reported by Feeney and colleagues, however (Feeney et al. 2013). Further research into the impact of traumatising events at different lifestages on later-life brain health is warranted to explain these inconsistencies. It is worth considering that participants had to have capacity to provide consent to be included in NICOLA so those with serious cognitive impairment were by design excluded from the outset, biasing study results. However given that scores on the Mini-Mental State Examination included a cognitively impaired range it is unlikely that NICOLA failed to capture those with cognitive impairment.

We also considered whether the effect of Troubles exposure and age on memory are confounded in our retrospective yet cross-sectional analysis because of “critical” or “sensitive” period effects”. We controlled for demographic, health, and social variables in the current analysis, and inspected levels of multicollinearity (which was nowhere found to be a problem), and the effects of Troubles variables on memory outcomes is nonetheless observed. This association could be explained with recourse to the resilient survivor cohort effect described previously (Barel et al. 2010)–i.e. that those who survive traumatic exposure (and be more likely to be recruited into cohorts) are higher in trait resilience, which mitigates later risk of morbidity, a form of collider stratification bias (Richiardi et al. 2019). These findings may also be driven, in part, by pre-existing differences in memory function; people with better memory may recall more events from their lives, just as they recall more words in a memory test.

We focus on traumatic exposure in the current analysis, rather than the psychological effects of such traumatic exposure, which are also associated with cognitive decline (Cohen et al., 2013). Post-traumatic Stress Disorder (PTSD) patients underperform in a range of cognitive domains, most notably memory, which seems to be driven by hippocampal volume differences between PTSD patients and healthy controls (Bremner and Narayan 1998). Disentangling the effects of traumatic exposure and related psychological impacts thereof upon cognitive functioning has proven difficult, historically, and longitudinal data would be required to comprehensively understand their relative impacts, something that can be done once later (planned) waves of NICOLA are completed. It will also be clarifying to consider the potential epigenetic mediators of the association between traumatic exposure and cognitive outcomes in the Northern Irish population, as is suggested by work in other populations (Cecil et al. 2016; Marzi et al. 2018).

At high levels of social activity engagement, Troubles Exposure exerted little effect on Memory, but at low levels of social activity engagement, those with higher levels of Troubles exposure outperformed those with lower levels of Troubles exposure. Kodesh had previously suggested that social activity engagement might offset the negative impact of traumatic exposure on dementia risk (Kodesh et al. 2019), but instead here we find that it there is no such negative impact to offset. Instead, it seems that for individuals who faced high levels of exposure to the Troubles, and thus high exposure to traumatic events during adulthood, memory functioning was preserved, possibly due to a resilience effect. This may have created a ceiling effect such that less variance was left for social activity engagement to account for–in individuals with lower levels of exposure to the Troubles, having a high level of social activity engagement mattered such that it was associated with better memory. Parsing the chain of risk events leading to positive outcomes in later life would help to determine the reality of the health impact of the Troubles, using a lifecourse epidemiological perspective to do so (Kuh et al. 2003).

Further inspiration can be taken from the life-course epidemiological perspective in relation to the “critical” or possibly “sensitive” period for cognitive decline; understanding precisely when events such as traumatic exposures can yield the largest effect on later-life cognitive functioning, by group comparison based on age at the peak of the Troubles, would also contribute to our understanding of this association. We had the data available to conduct this. Here our hypothesis was that traumatisation at different age groups (measured as age at self-rated worst point of the Troubles) would differentially impact memory functioning, yielding an effect of age at self-rated worst point of the Troubles upon memory functioning later in life. If, for instance, young adulthood is a critical or sensitive period for cognitive decline, then those participants who were young adults at the (self-rated) peak of the Troubles would fare worst in their later-life memory functioning. IF, on the other hand, middle adulthood were the critical or sensitive period, then those who were middle-aged during the self-rated peak of the Troubles would fare the worst.The results of our analyses (see Supplementary Materials 2) did not indicate that age at the self-rated worst point of the Troubles was predictive of memory functioning, nor did it moderate the impact of Troubles Exposure on memory functioning. Further research may be necessary to identify precisely the critical period for traumatic exposure during adulthood in relation to later-life cognitive functioning–from the current results this critical period does not appear to be during early/middle adulthood (our participants would have been aged 19–46 during the peak of the Troubles).

There are notable strengths worth highlighting in relation to the current study. NICOLA is a population representative study, with a large sample size and considerable sampling effort. A flexible statistical methodology was used which optimised the use of data despite the incomplete nature of the dataset. Data pertaining to a large, nationally representative group who were largely exposed to a period of civil unrest is potentially of use to life-course epidemiologists interested in broadening their frame of reference from childhood to early adulthood adversity. Relatedly, a strength of the current study is the exploration of traumatic exposure in a sample of non-military participants. This type of research is valuable to increase the heterogeneity of trauma research, which predominantly focuses on veterans and prisoners of war, meaning that women are under-represented (Schuitevoerder et al. 2013).

A limitation of the study is the cross-sectional nature of the data (although because of the retrospective nature of the questions, it is clear that Troubles Exposure temporally precedes social activity engagement and later-life memory functioning). Further, we cannot comment on the temporal nature of the relationship between social activity engagement and memory functioning, since these two were measured cross-sectionally, and it is possible that some reciprocal causation is indeed in effect here. The NICOLA study is a prospective cohort study so longitudinal data will be available in the near future for this sample. Re-exploration of our current research question with longitudinal data will be critical, since the impact of trauma over time may have a cumulative effect on cognitive function in later life (Schuitevoerder et al. 2013). Taking into account other potential moderators of the relationship between experience in the Troubles and health outcomes, such as conflict-related deprivation (O'Neill et al. 2015; O'Connor and O'Neill 2015), is also necessary.

To conclude, we report a moderating effect of social activity engagement and Troubles exposure on memory functioning in later life among those living in NI. Overall, those with a higher level of Troubles exposure did best on memory functioning, although there was no such effect among those with high levels of social activity engagement. Further research is required to fully elucidate the Troubles legacy as it relates to decline in later life in this cohort, and to identify particularly at-risk groups therein.

## Supplementary Information

Below is the link to the electronic supplementary material.

Supplementary file1 (DOCX 17 KB)
