# Supplementary material for: Exposure to the troubles in Northern Ireland, memory functioning, and social activity engagement: results from NICOLA
Source: Eur J Ageing. 2022 Feb 10;19(4):1099–109. doi: 10.1007/s10433-022-00683-5 (PMC9729674; doi:10.1007/s10433-022-00683-5)
Supplement: Supplementary file 1 — Supplementary file1 (DOCX 17 KB) [file 10433_2022_683_MOESM1_ESM.docx]

Supplementary Materials

Supplementary Material 1: Measurement Model for Troubles Exposure as a single latent factor, with Memory as a second latent factor.

In this model, Troubles Exposure had three indicators: Death (items of Q69 in Table 1), Injury (items of Q70 in Table 1), Witness (items of Q71 in Table 1). Memory had the same four indicators as were presented in the initial measurement model (Immediate Recall 1 and 2, Delayed Recall, Animal Naming). This model converged after 37 iterations and demonstrated good fit, χ^2^_49_ = 132, CFI = 0.99, TLI = 0.99, RMSEA = 0.038 (CI_90_ = 0.033, 0.044), SRMR = 0.043. All items had high factor loadings (see Table S1).

Table S1. Measurement Model with Troubles Exposure and Memory (latent factors included in Structural Model, in Results).

|  | Factor Loading | Standard Error | Z | p |
| --- | --- | --- | --- | --- |
| **Troubles Exposure** |  |  |  |  |
| Death | 0.702 | 0.015 | 41.136 | <.001 |
| Injury | 0.872 | 0.018 | 50.435 | <.001 |
| Witness | 0.640 | 0.031 | 37.246 | <.001 |
| **Memory** |  |  |  |  |
| Immediate Recall 1 | 0.818 | 0.020 | 72.299 | <.001 |
| Immediate Recall 2 | 0.881 | 0.021 | 80.496 | <.001 |
| Delayed Recall | 0.852 | 0.027 | 76.757 | <.001 |
| Animal Naming | 0.509 | 0.122 | 23.423 | <.001 |

Supplementary Materials 2: Does Age at Troubles moderate the impact of Troubles Exposure on Social Activity Engagement?

First a measurement model was conducted with Troubles Exposure, Memory, and a latent Interaction term composed of products between Troubles Exposure items (Death, Injury, Witness) and Age at Troubles (all mean-centred) as latent variables. In this measurement model, which converged normally after 46 iterations, fit was good, χ^2^_32_ = 151, CFI = 0.99, TLI = 0.99, RMSEA = 0.025 (CI_90_ = 0.021, 0.029), SRMR = 0.033, and all factor loadings were above 0.509 (they were 0.679, 0.832, and 0.635 for the three indicators in the latent Interaction term, respectively). As such the structural component was added in. The first SEM conducted contained no interaction term but Age at Troubles as a predictor, alongside Troubles exposure, and the same covariates as used above (age, gender, education, depression). This model converged normally after 119 iterations and fit was good, χ^2^_43_ = 1059, CFI = 0.94, TLI = 0.92, RMSEA = 0.060 (CI_90_ = 0.057, 0.063), SRMR = 0.076. for reference AIC was 195647 and ssaBIC was 195817. In the structural component of this model it was shown that being younger, β = -.414, p<.001, male, β = .123, p<.001, less depressed, β = -.130, p<.001, more educated, β = .274, p<.001, having a higher level of Troubles Exposure, β = .055, p=.005, all were associated with better memory functioning. Age at the worst time of the Troubles was not associated with memory functioning, β = .01, p=.685. a second SEM was conducted which included an interaction term to represent the moderating effect of Age at the worst time of the Troubles on the association between Troubles Exposure and memory functioning, to check for potential “critical period” effects. This model converged normally after 86 iterations and fit was good, χ^2^_77_ = 1563, CFI = 0.92, TLI = 0.90, RMSEA = 0.054 (CI_90_ = 0.052, 0.057), SRMR = 0.087. Fit was worse than the first model (AIC = 203034; ssaBIC = 203428, relative to AIC =195647 and ssaBIC 195817 above; chi squared difference = 504.36, df difference = 34, p<.001). Parameters are outlined in supplementary Table 2 but showed the same pattern of results as in the preceding model. Neither age at the worst time of the Troubles nor the interaction between Troubles Exposure and age at the worst time of the Troubles had a significant association with memory.

Supplementary Table 2. SEM with Troubles Exposure, Age at worst time of Troubles, and Interaction Term as predictors (with covariates), using maxmum likelihood estimator and bootstrapped standard errors.

| Predictor | Beta | SE | Z | P | CI_95_ |
| --- | --- | --- | --- | --- | --- |
| Age | -0.414 | 0.002 | -27.361 | <.001 | -.063, -.054 |
| Gender | 0.123 | 0.031 | 9.710 | <.001 | .244 - .367 |
| Depression | -0.130 | 0.025 | -7.639 | <.001 | -.239 - -.142 |
| Education | 0.274 | 0.024 | 21.541 | <.001 | .479 - .575 |
| Troubles Exposure | 0.055 | 0.025 | 2.721 | .007 | .018 - .117 |
| Age at Worst of Troubles | 0.017 | 0.041 | 0.518 | 0.605 | -.059 - .106 |
| Troubles Exposure * Age at Worst of Troubles | -0.015 | 0.046 | -.410 | .682 | -.107 - .077 |
